# Can indwelling pleural catheters provide additional benefits in elderly heart failure patients with pleural effusion? A real-world retrospective multicenter analysis

**DOI:** 10.3389/fcvm.2026.1680099

**Published:** 2026-04-20

**Authors:** Yinghua Wang, Yan Jin, Hui Li, Qi Guo, Qingqi Ji, Yan Pan, Min Zhang

**Affiliations:** 1 Department of Cardiology, Shanghai Chest Hospital, Shanghai Jiao Tong University School of Medicine, Shanghai, China; 2 Department of Pharmacy, Shanghai Chest Hospital, Shanghai Jiao Tong University School of Medicine, Shanghai, China; 3 Department of Cardiovascular Medicine, Ruijin Hospital Luwan Branch, Shanghai Jiao Tong University School of Medicine, Shanghai, China; 4 Department of General Practice, Community Health Service Center of Caohejing Street, Shanghai, Xuhui District, China

**Keywords:** acute heart failure, elderly, guideline-directed medical therapy, pleural drainage, pleural effusion

## Abstract

**Background:**

Pleural effusion (PE) is a common presentation in patients with congestive heart failure. Evidence on the necessity of therapeutic pleural drainage (PD) remains conflicting, highlighting a gap in optimal care for patients. This study aimed to compare standard diuretic therapy (SDT) within guideline-directed medical therapy (GDMT) versus PD in elderly acute heart failure (AHF) patients with moderate PE.

**Methods and results:**

We conducted a real-world multicenter, retrospective observational cohort study in China. We screened patients within the age range of 60–100 years who were admitted to hospital with AHF and moderate pleural effusions between January 2014 and January 2024. Patients were divided into two groups: the PD group and the GDMT group. The primary and secondary endpoints were time to spontaneous pleurodesis and readmission rate, respectively. Of the 936 elderly AHF patients with moderate pleural effusion who were screened, 514 of them were included in final analysis. Time to spontaneous pleurodesis and time to discharge were shorter in the GDMT group than in the PD group (*P* = 0.001, *P* = 0.001). There were no differences in 90- and 180-day readmission rates between the two groups (hazard ratio (HR) 1.450, *P* = 0.063 and HR 1.383,*P* = 0.068).

**Conclusion:**

SDT within GDMT yielded comparable outcomes to pleural drainage in elderly AHF patients with moderate PE, with respect to effusion resolution and hospital length of stay, without increased risk of worsening renal function or electrolyte imbalance.

## Introduction

Congestion is the hallmark of acute heart failure (AHF). Increased fluid transfer develops due to increased capillary pressure from elevated venous outflow pressure and decreased lymphatic flow into central vessels in the setting of heart failure (1). Pleural effusion (PE) is another manifestation in patients with significant fluid overload and plays a prognostic role in AHF. Patients can present with shortness of breath, chest pain, and paroxysmal nocturnal dyspnea, although the severity of symptoms often correlates poorly with the volume of pleural effusion (2).

Medical treatment is the cornerstone of HF. Current guidelines recommend loop diuretics—such as intravenous loop diuretics, administered either as bolus or continuous infusion—to ameliorate the symptoms of fluid overload in AHF (3–5). Complete resolution of pleural effusions with diuretics may require several weeks, depending on their original size. For non-malignant PEs (NMPEs), unlike malignant PEs where clear guidance already exists, therapeutic pleural drainage (PD) is usually reserved for cases when pharmacological interventions such as diuretics fail or if the patient is significantly symptomatic. Removal of pleural fluid through therapeutic thoracentesis (TT) or indwelling pleural catheters (IPCs) accelerates the alleviation of patient symptoms.

To date, the REDUCE study is the only RCT that has evaluated the efficacy of IPCs in refractory NMPE compared with TT (2, 6, 7). Results showed no significant differences in symptom improvement between the IPC and TT approaches. Conversely, TT often requires additional invasive procedures compared with IPC (8, 9), which bear higher risks of pain, hemorrhage, and infection (10, 11). In addition, Glargaard et al. demonstrated that routine upfront thoracentesis did not increase the number of days alive and out of the hospital at 90 days compared with standard medical therapy (12). Conflicting evidence on the necessity of therapeutic pleural drainage indicates that a gap still exists in optimal care for this patient population.

It remains unclear whether a conservative strategy using guideline-directed medical therapy (GDMT) could yield comparable clinical outcomes to pleural drainage regarding the specific clinical resolution of the effusion, without imposing excessive renal burden or electrolyte disturbance. Therefore, we aimed to assess whether standard diuretic therapy (SDT) within GDMT is comparable to pleural drainage in elderly AHF patients with ultrasound-quantified medium pleural effusions. We focused on clinical outcomes, renal safety, and diuretic requirements in a real-world setting where anticoagulation was maintained.

## Methods

### Ethical statement

This retrospective study was conducted to assess whether standard pharmacological diuretic treatment is comparable to therapeutic pleural drainage. The trial was conducted in accordance with the Declaration of Helsinki and was approved by the Clinical Research Ethics Committees of Shanghai Jiao Tong University School of Medicine, Shanghai Chest Hospital (IS25070), Ruijin Hospital (LWEC2025021), and the Community Health Service Center of Caohejing, Xuhui District, Shanghai.

### Study design and procedures

Between 1 January 2014 and 1 January 2024, patients aged 60 years to 100 years with a primary diagnosis of AHF and moderate pleural effusion were enrolled in this study. Follow–up continued until 1 September 2024 through outpatient consultation or by telephone. Patients were divided into two groups depending on whether or not they underwent pleural drainage: the PD group and the GDMT group.

### Quantification of pleural effusion

Pleural effusion was diagnosed using chest radiography and ultrasound. On radiographs, when a curved upper border of the meniscus sign is between the second rib and the fourth rib in an upright posteroanterior view, it indicates the presence of a medium amount of pleural fluid; the upper border being above the second rib signifies a large amount of pleural effusion. Blunting of the lateral costophrenic angle usually indicates a slight amount of pleural fluid. Ultrasound, in combination with chest radiography, is more sensitive and is readily available for diagnosing and confirming the amount of effusion. Medium pleural effusion is defined as a fluid depth of 30–50 mm and a height of 70–110 mm by ultrasound (13–15).

### Study population and participants

The study screened elderly (60–100 years) patients admitted to the Cardiology Departments of Shanghai Chest Hospital, Ruijin Hospital Luwan Branch, Shanghai Jiao Tong University School of Medicine, and Community Health Service Center of Caohejing, Xuhui District, Shanghai, between January 2014 and January 2024. Individuals were eligible for enrollment if they had a diagnosis of acute decompensated heart failure with unilateral or bilateral pleural effusions documented by either chest radiography or ultrasound, defined as a moderate in volume.

If the patient had been admitted multiple times during the study period, only the first admission was considered valid for data collection.

PEs with underlying causes other than heart failure—such as lung cancer, infection, and liver cirrhosis—were excluded. Other exclusion criteria included history of intrathoracic procedures or open-heart surgery within 1 year and absence of intravenous diuretic utilization.

**Figure 1:**
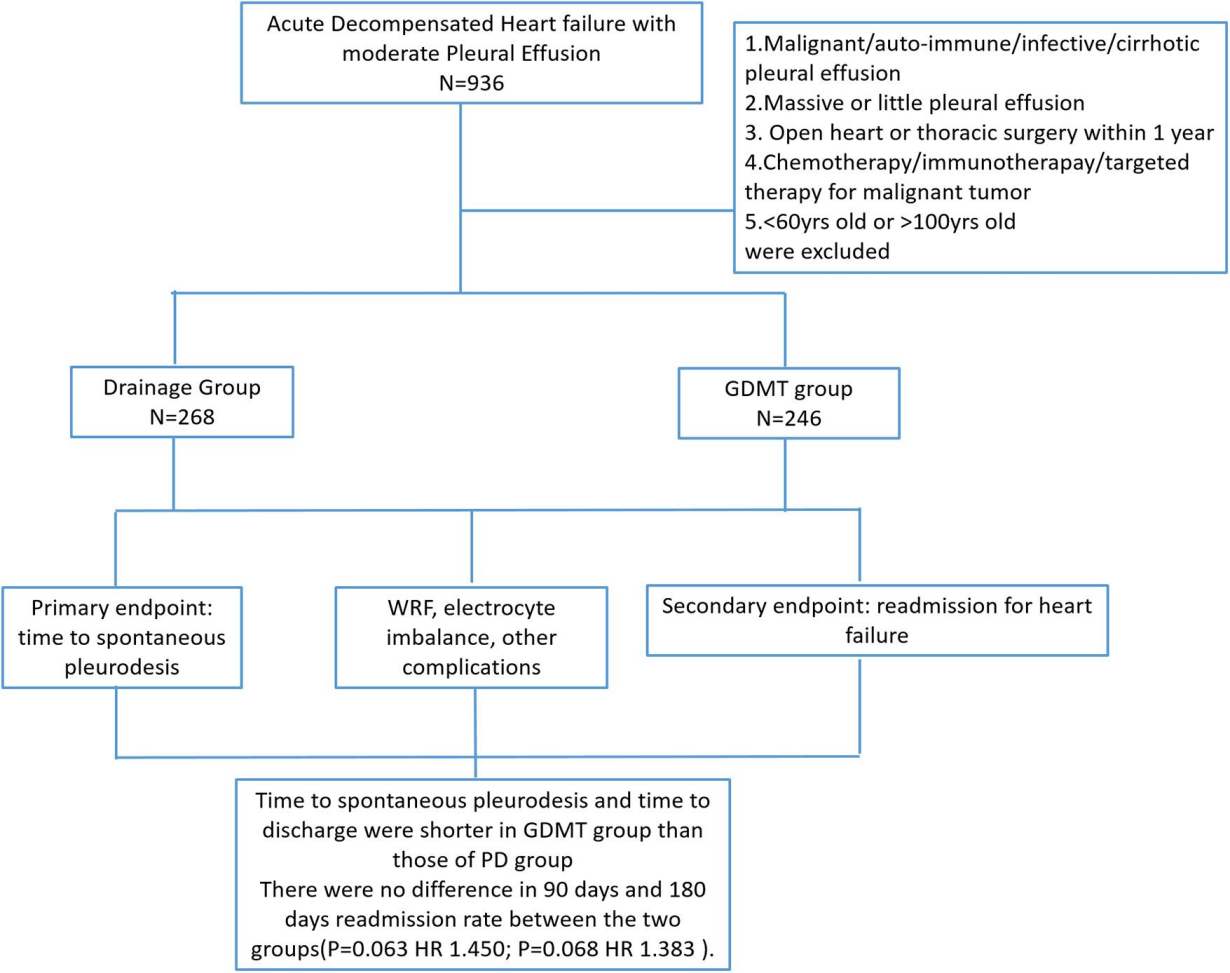
Patient flow diagram of the study.

A complete list of the inclusion and exclusion criteria is provided in Figure 1.

### Study intervention (medication and drainage)

Enrolled participants were divided into two groups depending on whether or not they underwent pleural drainage. PE drainage was performed according to the standard drainage procedures using pigtail catheters attached to a chest drainage device.

Standard GDMT medication included a low-sodium diet, fluid restriction, loop diuretics, angiotensin II receptor blocker (ARB)/neprilysin inhibitor (ARNI), evidence-based beta blockers, sodium-glucose Cotransporter-2 (SGLT-2) inhibitors, and mineralocorticoid receptor antagonist (MRA) (3). Loop diuretics form the backbone of treatment for AHF, though medication regimens are not standardized in guidelines, leaving the individualization to the physician. Intravenous administration was preferred for inpatient management, with later transition to oral formulation when the patient was stabilized and ready for discharge. Therefore, we only collected information of intravenously administered diuretics to facilitate quantifying the daily dose. Different types of diuretics were uniformly converted to furosemide, with the equivalent ratio of 2 for torsemide and 20 for bumetanide.

Other key medications that could enhance diuresis were also analyzed as part of the diuretic regimen, including nesiritide, a recombinant B-type natriuretic peptide (BNP) routinely used to reduce pulmonary capillary wedge pressure and improve dyspnea.

Diuretic resistance or poor response was assessed as being unresponsive to double-dose furosemide, requiring the use of more than two types of diuretics or requiring continuous intravenous infusion. Albumin infusion was administered when indicated to increase blood volume, correct hypoalbuminemia, or maintain normal colloid osmotic pressure. Moreover, both groups received standard therapy for acute heart failure.

Worsening renal function (WRF), similar to acute kidney injury (AKI), was defined as an increase in serum creatinine of ≥0.3 mg/dL (≥26.5 μmol/L) within 48 h, an increase in serum creatinine by 50% compared with the baseline within 7 days—meaning it is 1.5 times the original value or greater—or urine volume of <0.5 mL/kg/hour for 6 h (KDIGO 2012) (16).

The drainage was carried out once pleural effusion was diagnosed by X-ray and ultrasound upon admission, along with a suitable position determined by ultrasound. The drainage protocol involved capping the volume of drained fluid to a maximum of 800 mL per day to prevent occurrence of re-expansion pulmonary edema. All patients in the drainage group received indwelling pleural catheters. Indwelling pleural catheters were pigtail catheters—soft, flexible tube thinner than a pencil—that remained inside the chest and exited through the skin, continuously attached to a chest drainage device. The drainage procedure included the following steps: skin antisepsis with iodophor, local anesthesia, needle insertion, guiding wire placement, needle removal, catheter insertion, wire removal, drainage confirmation, and catheter fixation (2, 6).

### Clinical endpoints

The primary endpoint was time to achieving spontaneous pleurodesis. All patients were re-evaluated for confirmation of pleurodesis by ultrasound and X-ray. Pleurodesis was considered achieved if the following were fulfilled: (1) patients from either group showed no re-accumulation or persistence of pleural fluid, confirmed by radiography and ultrasound of the chest, showing sharp costophrenic angles in chest X-ray and less than 30 mm depth and 50 mm width in ultrasound; and (2) patients in the PD group had less than 50 mL of fluid drained on three consecutive occasions through the indwelling pleural catheter.

The secondary endpoint was the number of patients readmitted for heart failure after discharge. Follow-up was conducted by telephone and outpatient consultation after discharge.

### Statistical analysis

For the primary endpoint, we assumed an average time to spontaneous pleurodesis of 5 days. It was estimated that a total of 185 patients would provide the trial with 80% power, accounting for a 5% dropout rate. The upper boundary of the 90% confidence interval for the estimated mean between-group difference had to be 5 days to meet the criterion.

All statistical analyses were performed using SPSS 27.0 software (IBM Corp. Released 2017. IBM SPSS Statistics for Windows, Version 25.0. Armonk, NY: IBM Corp.) and R version 4.2.1 (CRAN; https://www.r-project.org/) for windows. Homogeneity of variance was tested to determine quantitative data distribution. When the distribution of variables was normal, independent samples Student's *t*-test was used to compare quantitative values between two independent groups, and continuous variables were expressed as mean (standard deviation). Non-normally distributed variables were presented as median with interquartile range (IQR), and the Mann–Whitney *U*-test was applied. Categorical variables were evaluated using the Chi-squared (*χ*^2^) test. When marginal observed frequencies were smaller than 5, Fisher's exact test was used. Cox proportional hazards regression mode was adopted to identify differences between the two groups in 90- and 180-day re-hospitalization for acute heart failure. A *P* value of less than 0.05 was considered statistically significant.

## Results

### Baseline clinical characteristics

Between January 2014 and January 2024, 3,070 patients were admitted to any of the three study centers with AHF. Of these, 936 patients fell within the age range and had moderate pleural effusion. After screening, 422 patients were excluded for a variety of reasons (Figure 1), leaving 514 patients for final analysis.

Baseline characteristics were generally balanced between the two groups (Table 1). The mean age of this cohort study was 74.92 ± 8.18 years, and patients were predominantly males. Among them, 129 patients had a history of ischemic heart disease, 146 had atrial fibrillation, 182 had heart valvular diseases, and others had primary cardiomyopathy. Results of propensity score matching are presented in Supplementary Table 1.

**Table 1:** Baseline in-hospital characteristics between the two groups.

Characteristics	GDMT group	Drainage group	*P*
Gender (M/F)	168/78	178/90	0.651
Age	75.66 ± 8.31	74.25 ± 8.02	0.051
Medical history			0.362
IHD	56	73	
HVD	87	95	
A-Fib	78	68	
Pericardial effusion	106	113	0.832
Serum albumin (g/L)	30.00 (9.00)	29.00 (4.00)	0.55
Primary NT-proBNP	19,000 (8,695)	20,700 (15,900)	0.087
Max NT-proBNP	21,000 (8,970)	35,000 (13,500)	0.098
Discharge NT-proBNP	2,518.54 ± 1,871.20	2,533.32 ± 1,788.67	0.927
Echo-LVEF (%)	48 (23)	42 (21)	0.475
Echo-LAD (mm)	46.08 ± 6.30	44.97 ± 8.49	0.096
Echo-LVDd (mm)	51.00 (14)	53.00 (10)	0.081
Echo-PHT (mmHg)	46.17 ± 13.96	48.40 ± 15.33	0.086
SBP	113.73 ± 11.95	112.70 ± 12.40	0.339
Breathlessness	246	268	
NYHA (2/3/4)	36/178/32	33/211/24	0.208
Unilateral moderate PE	246	198	
Bilateral moderate PE	0	70	
GDMT, guideline-directed medical therapy; M/F, male/female; SBP, systolic blood pressure; PE, pleural effusion; NYHA, New York Heart Association Functional Classification; NT-proBNP, pro-brain natriuretic peptide (pg/L); LVEF, left ventricular ejection fraction (%); LAD, left atrial diameter (mm); LVDd, left ventricular diastolic diameter; PHT, pulmonary hypertension; IHD, ischemic heart disease; HVD, heart valvular disease; A-Fib, atrial fibrillation			

Several baseline differences were noted between the two groups. Of the 514 elderly AHF patients with moderate pleural effusion, no differences were found in age, gender, medical history, clinical presentation, NYHA (New York Heart Association), pericardial effusion, unilateral or bilateral pleural effusion, primary NT-proBNP, maximum NT-proBNP, discharge NT-proBNP, and left ventricular ejection fraction (LVEF), left ventricular diastolic diameter (LVDd), left atrial diameter (LAD), and pulmonary hypertension (PHT) parameters, as noted through echocardiography.

### Hospital procedures and medical therapy

Hospital procedures, therapies, and complications are summarized in Table 2. Diuretic regimen and loop diuretic dosages were not significantly different between the two groups. Nevertheless, dosage of albumin was higher in the drainage group (*P* = 0.001). The incidence of complications such as electrolyte imbalance and WRF were similar between the two groups. Time to spontaneous pleurodesis was shorter in the GDMT group (*P* = 0.001), while time to discharge was longer in the drainage group (*P* = 0.001). Moreover, readmission rates did not differ between the two groups. Result after propensity score matching are shown in Supplementary Table 2. Drainage-related complications are shown in Supplementary Table 3.

**Table 2:** Treatment, complication, and endpoint between the two groups.

Characteristics	GDMT group	Drainage group	*P*
Time to discharge (day)	9.00 (5)	11.00 (11)	0.001
Diuretic regimen			
1	90	99	0.053
2	8	24	
3	128	123	
4	20	22	
Dosage of diuretics (mg/day)	23.75 (25)	22.36 (19)	0.128
WRF	40	59	0.098
Electrolyte imbalance	40	60	0.080
Complication of drainage	0	13	
Dosage of albumin (T/10 g)	0.00 (0)	2.00 (3)	0.001
Ventilation	134	127	0.109
Readmission	88	83	0.248
Time to spontaneous pleurodesis (day)	9.00 (5)	10.00 (9)	0.001

GDMT, guideline-directed medical therapy.

Diuretic Regimen: regimen 1 refers to intravenous bolus injection of loop diuretic; 2 as continuous intravenous infusion of loop diuretic; 3 as continuous intravenous infusion of loop diuretic and nesiritide; 4 refers to loop diuretic given in both bolus injection and continuous infusion as well as infusion of nesiritide.

WRF, worsening renal function; T/10 g, total albumin dosage/10 g.

### 90- and 180-day follow-up

**Figure 2:**
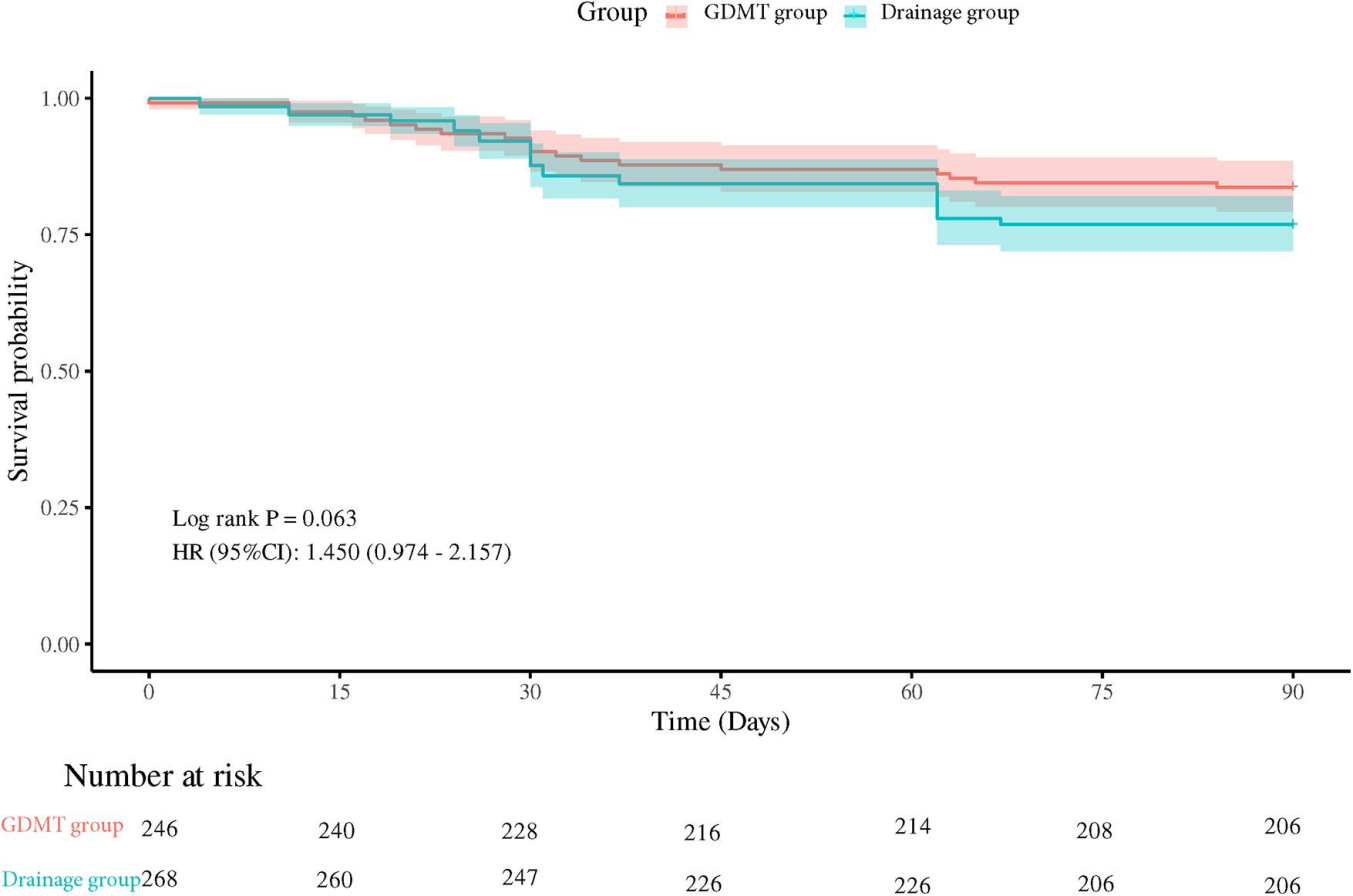
Ninety-day readmission rates between the two groups.

**Figure 3:**
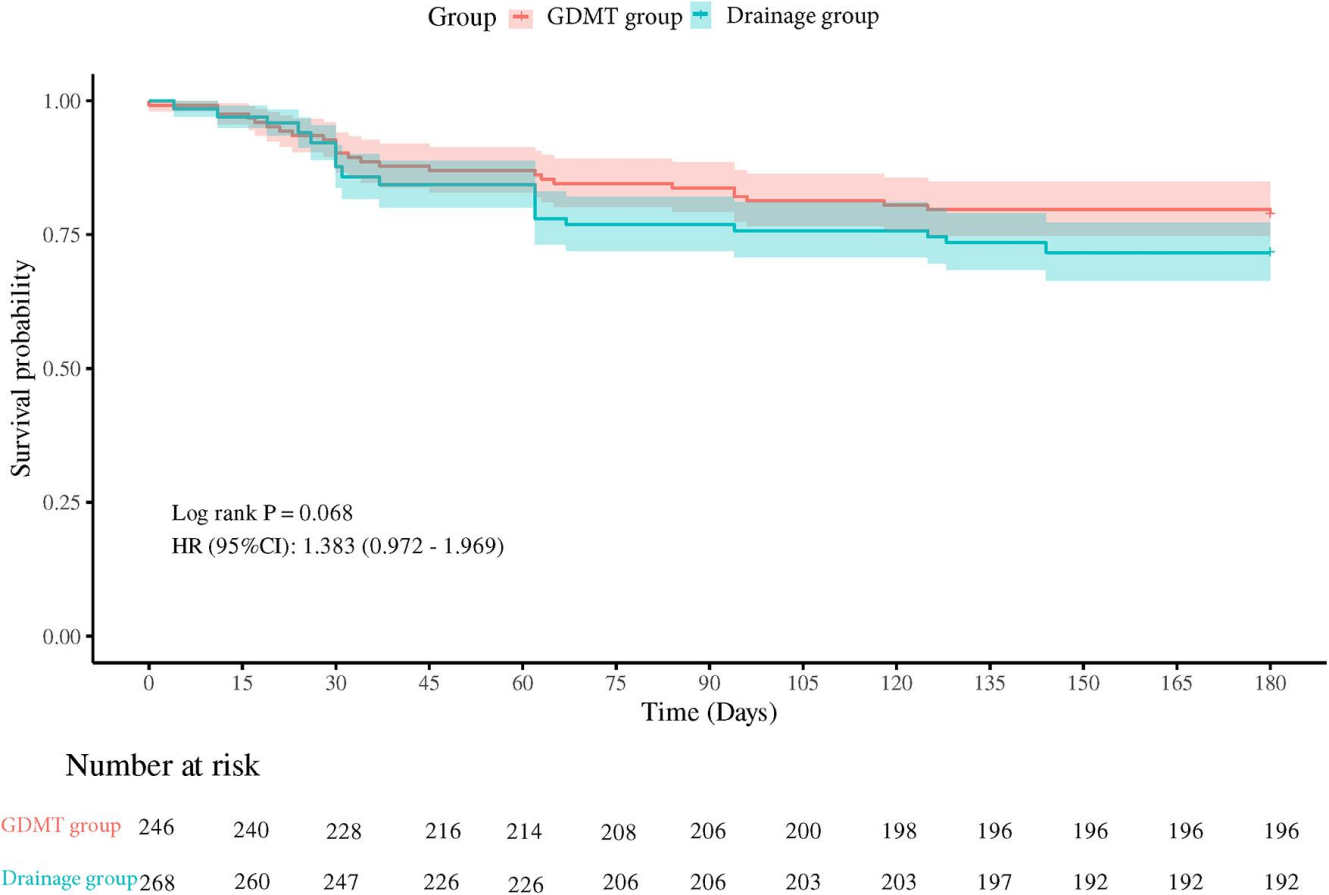
One hundred eighty-day readmission rates between the two groups.

We used Cox proportional hazards model to identify the difference between the two groups in first readmission after discharge (Figures 2, 3). At both 90- and 180-day follow-up, readmission rates were similar (hazard ratio (HR): 1.450, 95% CI 0.974–2.157, *P* = 0.063; HR: 1.383, 95% CI 0.972–1.969, *P* = 0.068). Moreover, age and primary disease were significant predictors for readmission due to heart failure according to multiple regression analysis (Table 3).

**Table 3:** Multiple regression analysis of predictors in readmission.

Variables in equation									
	Variables	*B*	S.E.	Wals	df	Sig.	Exp (B)	95% CI for Exp(B)	
								Lower upper	
Step1^a^	Age	0.024	0.012	4.100	1	0.043	1.025	1.001	1.049
	Time to discharge (day)	0.014	0.048	0.080	1	0.778	1.014	0.922	1.114
	Treatment	−0.308	0.203	2.315	1	0.128	0.735	0.494	1.093
	Max NT-proBNP	0.000	0.000	0.854	1	0.356	1.000	1.000	1.000
	LVEF	0.001	0.008	0.007	1	0.933	1.001	0.986	1.016
	Dosage of diuretics (mg/day)	−0.005	0.003	2.099	1	0.147	0.995	0.989	1.002
	Time to spontaneous pleurodesis (day)	0.004	0.051	0.006	1	0.939	1.004	0.908	1.110
	Primary disease	−0.213	0.090	5.584	1	0.018	0.808	0.677	0.964
	Constant	−1.365	1.143	1.425	1	0.233	0.255		

Treatment: sole GDMT or drainage + GDMT.

NT-proBNP, pro-brain natriuretic peptide (pg/L); LVEF, left ventricular ejection fraction (%).

^a^ Variables entered in step 1: age, time to discharge (day), treatment, Max NT-proBNP, LVEF, dosage of diuretics (mg/day), time to spontaneous pleurodesis (day), and primary disease.

## Discussion

Current treatment recommendations for patients with heart failure and pleural effusion are ambiguous. Standard diuretic therapy within GDMT is the cornerstone of AHF management (1, 8, 9, 17–20). For patients with significant fluid overload such as pleural effusion, therapeutic drainage may be considered and should help shorten time to discharge (6). This study tested the hypothesis that standard pharmacological therapy yields comparable clinical outcomes to therapeutic pleural drainage plus medication in elderly AHF patients with medium volume of pleural effusions.

Our findings align with the recently published TAP-IT trial, the first randomized controlled study to compare therapeutic thoracentesis with medical therapy in AHF. TAP-IT found that upfront thoracentesis did not improve the primary outcome of “days alive out of the hospital” or reduce 90-day mortality. Similarly, our retrospective analysis suggests that for medium-sized effusions, SDT within GDMT achieved results comparable to pleural drainage in terms of PE resolution and length of hospital stay. While the TAP-IT trial provides high-quality randomized evidence against routine drainage for general outcomes, our study adds further details supporting a conservative approach, specifically for medium-volume pleural effusions. We confirm that sparing elderly patients the trauma of invasive drainage does not prolong hospitalization or compromise resolution of the effusion. Moreover, TAP-IT relied on subjective visual estimation of effusion size (“non-negligible”) rather than standardized quantification, and mandated the interruption of anticoagulant therapy prior to the procedure. Consequently, questions remain regarding the optimal management of strictly quantified “medium-volume” pleural effusions, particularly in elderly patients with high thrombotic risks, where withholding anticoagulation is undesirable.

Our study compared pleural drainage plus diuretic therapy with only diuretic therapy in elderly AHF patients with moderate pleural effusion. The primary endpoint indicated that the invasive procedure did not significantly shorten the time to spontaneous pleurodesis or time to discharge compared with the GDMT group. Discharge criteria generally included symptom improvement, reduction in NT-proBNP, and ultrasound and X-ray confirmation of no re-accumulation or persistence of pleural fluid. The longer length of stay observed in the drainage group may reflect factors unrelated to clinical recovery, such as catheter management logistics. Accordingly, differences in length of stay should not be interpreted as proof that one strategy accelerates recovery. Moreover, we observed greater albumin use in the drainage group despite similar baseline serum albumin levels of 29.44 ± 3.80 g/L as shown in Table 1, while the usage of diuretics was comparable between the groups. This might reflect the loss of preload due to drainage, even though the volume drained was capped at 800 mL per day. Previous studies have reported lower concentrations of serum albumin in drainage groups, probably explained by the greater volume of drainage compared with the TT group. Thus, when making decisions between IPC and TT, clinicians should weigh the benefit of avoiding additional procedures against the risk of undesired adverse effects such as hypoalbuminemia (1, 8, 9). Albumin infusion could correct hypoalbuminemia and may help mobilize fluid from the interstitial and pleural spaces back into the intravascular compartment, thereby enhancing the efficacy of loop diuretics and potentially improving congestion symptoms (21, 22). Meanwhile, pleural drainage did not decrease the number of readmissions during 180-day follow-up or reduce the interval between discharge and first readmission.

Previous studies have found that poor diuretic response is associated with more advanced heart failure, renal impairment, diabetes, atherosclerotic disease, and in-hospital worsening heart failure, and that it predicts mortality and readmission due to heart failure (23–26). For patients who demonstrate diuretic resistance or remain hemodynamically unstable, it is reasonable for physicians to consider more aggressive therapy. In our study, we compared loop diuretic dose, route of administration, and use of albumin and anti-diuretic hormone agent between the two groups to illustrate the extent of fluid overload and diuretic resistance or poor response. Different diuretics were converted to furosemide to evaluate the actual daily dose of diuretics in our study. Our two groups demonstrated generally balanced baseline characteristics, such as types and dosage of diuretics, as well as incidence of electrolyte imbalance, WRF, and ventilation. Notably, we compared diuretic resistance in patients, defined as more than two types diuretics used or the requirement for continuous intravenous infusion of loop diuretics, and we found no differences between the two groups on level of diuretic response.

To our knowledge, this is the first real-world multicenter retrospective study to focus on elderly heart failure patients with moderate pleural effusion. Previous research has largely consisted of case series or small-scale retrospective cohort studies. They also included pleural effusions of mixed etiology, which may lead to risk of bias. We assessed the extent of diuretic resistance between the two groups by comparing the number of diuretics prescribed. Different loop diuretics were converted to furosemide so that a mean daily dose could be calculated (27).

However, our study still has a few limitations. First, there are still no objective decision criteria for drainage and timing of intervention. This was one reason why we chose to focus on patients with a medium volume of pleural effusion—a population for whom the clinical choice between pharmacotherapy alone and a more aggressive procedure is often ambiguous. It is important to acknowledge that pleural drainage was determined by cardiologists’ personal preference and experience. This individualized clinical judgment aligns with a predominant pattern in secondary care institutions, which often prioritize less invasive interventions, particularly given the emergence of more potent diuretics offering effective management for fluid overload. Thus, further randomized controlled trials are needed to provide solid evidence. Second, there is a lack of standardized guidelines for quantifying pleural effusion. Pleural effusion was diagnosed by X-ray and chest ultrasound upon admission in our study. The volume of fluid drained varied among patients and did not necessarily correlate with the actual amount of pleural effusion. The volume was capped by how well heart failure patients tolerated the drainage. Third, we did not capture patient-reported outcomes or standardize dyspnea/functional status measurements, limiting conclusions about symptomatic benefits. As this was a retrospective study, such information was not recorded in routine clinical practice. In heart failure patients, suffering can be partially alleviated by removing the overload fluid, which in turn improves daily functioning. Therefore, we chose the time to spontaneous pleurodesis as a surrogate primary endpoint over patient-reported outcomes, reasoning that radiographic resolution provides objective evidence of efficacy. Finally, like all retrospective studies, the patient population for this investigation was relatively small and potential misclassification bias cannot be completely excluded. Residual confounding may persist even when measured baseline characteristics appear similar.

Taken together, outcomes were similar between GDMT alone and pleural drainage plus medical therapy with respect to effusion resolution and hospital length of stay in elderly AHF patients with moderate pleural effusion, without increased risk of WRF or electrolyte imbalance. These findings are hypothesis-generating and support the need for prospective studies to identify patient subgroups that may benefit from invasive drainage, while carefully weighing procedural risks and resource utilization against conservative medical management.

## Data Availability

The original contributions presented in the study are included in the article/Supplementary Material, further inquiries can be directed to the corresponding author.

## Supplementary material

Supplementary Table 1 Baseline in-hospital characteristics between the two groups after PSM.

Supplementary Table 2 Treatment, complication and endpoint between the two groups after PSM.

Supplementary Table 3 Complications of drainage.

Supplementary Figure 1 Flowchart of timeline.
