# Supplementary material for: Can indwelling pleural catheters provide additional benefits in elderly heart failure patients with pleural effusion? A real-world retrospective multicenter analysis
Source: Front Cardiovasc Med. 2026 Apr 20;13:1680099. doi: 10.3389/fcvm.2026.1680099 (PMC13136148; doi:10.3389/fcvm.2026.1680099)
Supplement: Supplementary Table 1 — Baseline in-hospital characteristics between the two groups after PSM. [file Table1.docx]

| **Supplementary Table 1. Baseline in-hospital characteristics between the two group in PSM** | | | |
| --- | --- | --- | --- |
| characteristics | GDMT group | Drainage group | P |
| Gender（M/F） | 130/73 | 145/58 | 0.137 |
| Age | 74.95±8.29 | 74.95±8.29 | 0.82 |
| Medical History |  |  | 0.072 |
| IHD | 46 | 61 |  |
| HVD | 72 | 70 |  |
| A-Fib | 25 | 32 |  |
| Pericardial effusion | 90 | 94 | 0.765 |
| Serum albumin(g/L) | 30.00(9.00) | 29.00(4.00) | 0.98 |
| Primary NT-proBNP | 18850 (8032) | 20000(12422) | 0.611 |
| Max NT-proBNP | 21000(9510) | 22070(14000) | 0.967 |
| Discharge NT-proBNP | 2519.51±1882.06 | 2501.95±1897.64 | 0.925 |
| Echo-LVEF(%) | 49(24) | 43(21) | 0.912 |
| Echo-LAD(mm) | 46(8) | 43(3) | 0.434 |
| Echo-LVDd(mm) | 50.00(14) | 53.00(10) | 0.548 |
| Echo-PHT(mmHg) | 47.59±13.94 | 49.01±16.05 | 0.342 |
| SBP | 111.98±11.41 | 109.93±12.07 | 0.080 |
| Breathlessness | 203 | 203 |  |
| NYHA(2/3/4) | 29/153/21 | 33/143/27 | 0.510 |
| Unilateral moderate PE | 203 | 158 |  |
| Bilateral moderate PE | 0 | 45 |  |
| GDMT: Guideline-directed medical therapy  M/F: male/female SBP: systolic blood pressure PE: pleural effusion  NYHA-New York Heart Association Functional Classification  NT-proBNP—pro-brain natriuretic peptide (pg/L)  LVEF: left ventricular ejection fraction(%); LAD: left atrial diameter(mm); LVDd: left ventricular diastolic diameter; PHT: pulmonary hypertension; mm:millimeter; mmHg: millimetres of mercury  IHD: Ischemic heart disease, HVD: heart valvular disease, A-Fib: Atrial fibrillation | | | |
