# Supplementary material for: Can indwelling pleural catheters provide additional benefits in elderly heart failure patients with pleural effusion? A real-world retrospective multicenter analysis
Source: Front Cardiovasc Med. 2026 Apr 20;13:1680099. doi: 10.3389/fcvm.2026.1680099 (PMC13136148; doi:10.3389/fcvm.2026.1680099)
Supplement: Supplementary Table 2 — Treatment, complication and endpoint between the two groups after PSM. [file Table2.docx]

| **Supplementary Table 2. Treatment, complication and endpoint between the two group in PSM** | | | |
| --- | --- | --- | --- |
| characteristics | GDMT group | Drainage group | P |
| Time to discharge(day) | 8.00(5) | 9.00(11) | 0.001 |
| Diuretic Regimen 1 | 71 | 76 | 0.166 |
| 2 | 7 | 16 |  |
| 3 | 109 | 93 |  |
| 4 | 16 | 18 |  |
| Dosage of diuretics  (mg/d) | 39.00±43.03 | 32.79±32.48 | 0.101 |
| WRF | 31 | 47 | 0.058 |
| Electrolyte imbalance | 31 | 45 | 0.098 |
| Complication of drainage | 0 | 13 |  |
| Dosage of albumin (T/10g) | 0(1) | 2(5) | 0.001 |
| Ventilation | 110 | 92 | 0.091 |
| Re-admission | 73 | 65 | 0.463 |
| Time to Spontaneous pleurodesis (day) | 8(5) | 10(10) | 0.001 |

GDMT: Guideline-directed medical therapy

Diuretic Regimen: regimen 1 refers to intravenous bolus injection of loop diuretic; 2 as continuous intravenous infusion of loop diuretic; 3 as continuous intravenous infusion of loop diuretic and nesiritide; 4 refers to loop diuretic given in both bolus injection and continuous infusion as well as infusion of nesiritide

WRF: Worsening renal function

mg /d: miligram/day

T/10g: total albumin dosage/10g
