# Supplementary material for: Can indwelling pleural catheters provide additional benefits in elderly heart failure patients with pleural effusion? A real-world retrospective multicenter analysis
Source: Front Cardiovasc Med. 2026 Apr 20;13:1680099. doi: 10.3389/fcvm.2026.1680099 (PMC13136148; doi:10.3389/fcvm.2026.1680099)
Supplement: Supplementary Table 3 — Complications of drainage. [file Table3.docx]

Supplementary Table 3.

| Complication of drainage | N | Managements |
| --- | --- | --- |
| Skin bleeding | 1 | Re-suture for fixation |
| Pheumothorax | 1 | Converted to/Re-attached to a closed chest drainage device |
| Poor drainage | 2 | Re-cannulation |
| Severe pain in drainage site | 5 | Pain relief therapy, such as paracetamol |
| Tube displacement | 4 | Re-cannulation |
