# Supplementary figures and images for: Can indwelling pleural catheters provide additional benefits in elderly heart failure patients with pleural effusion? A real-world retrospective multicenter analysis
Source: Front Cardiovasc Med. 2026 Apr 20;13:1680099. doi: 10.3389/fcvm.2026.1680099 (PMC13136148; doi:10.3389/fcvm.2026.1680099)

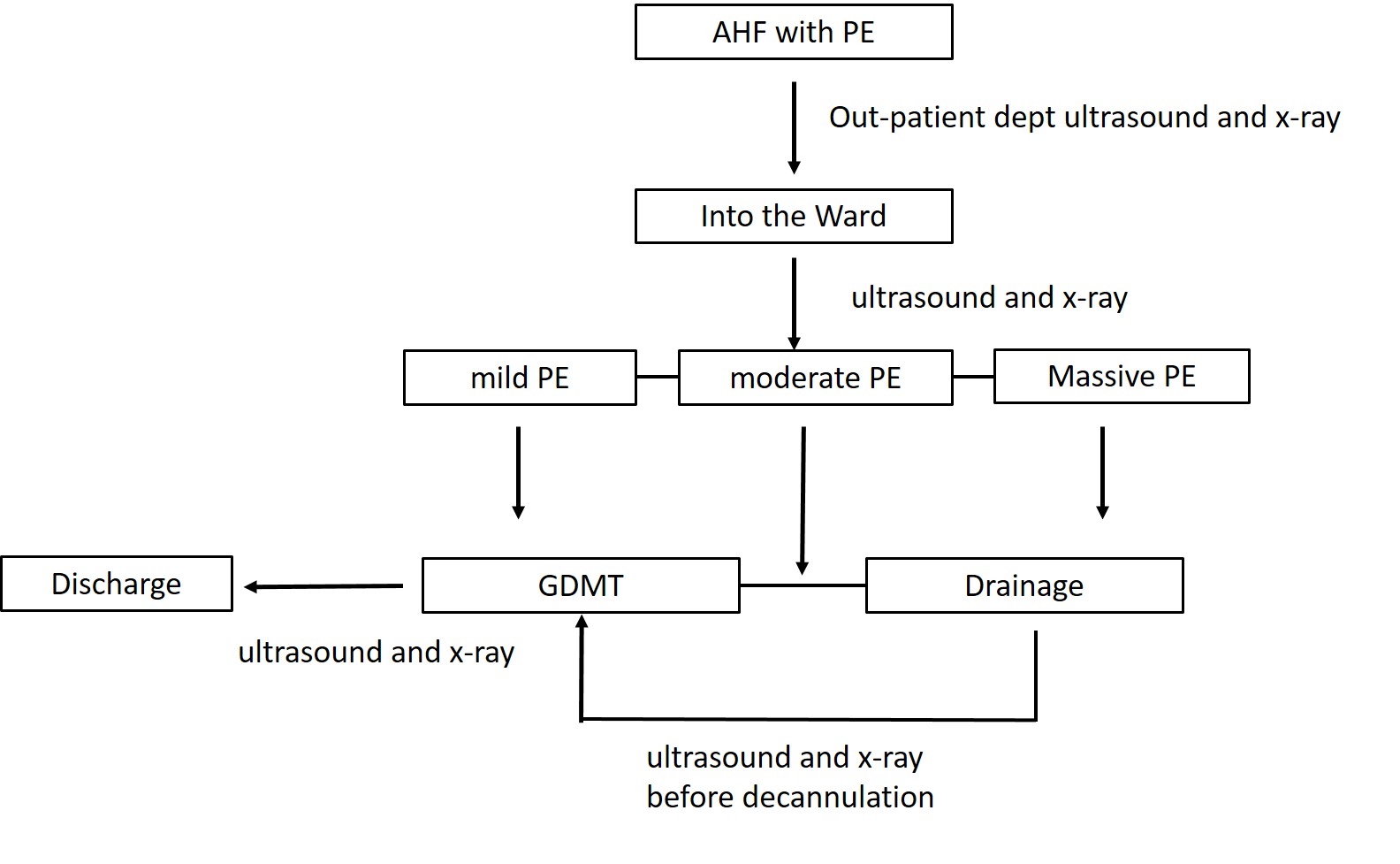

Supplement: Supplementary Figure 1 — Flowchart of timeline. [file Image1.jpeg]
